# Gene Loss may have Shaped the Cnidarian and Bilaterian Hox and ParaHox Complement

**DOI:** 10.1093/gbe/evac172

**Published:** 2022-12-12

**Authors:** Bailey M Steinworth, Mark Q Martindale, Joseph F Ryan

**Affiliations:** Whitney Laboratory for Marine Bioscience, University of Florida, St Augustine, Florida 32080; Department of Biology, University of Florida, Gainesville, Florida 32611; Whitney Laboratory for Marine Bioscience, University of Florida, St Augustine, Florida 32080; Department of Biology, University of Florida, Gainesville, Florida 32611; Whitney Laboratory for Marine Bioscience, University of Florida, St Augustine, Florida 32080; Department of Biology, University of Florida, Gainesville, Florida 32611

**Keywords:** Hox, homeobox, cnidarian–bilaterian ancestor, Medusozoa, *Cassiopea xamachana*

## Abstract

Hox and ParaHox transcription factors are important for specifying cell fates along the primary body axes during the development of most animals. Within Cnidaria, much of the research on Hox/ParaHox genes has focused on Anthozoa (anemones and corals) and Hydrozoa (hydroids) and has concentrated on the evolution and function of cnidarian Hox genes in relation to their bilaterian counterparts. Here we analyze together the full complement of Hox and ParaHox genes from species representing all four medusozoan classes (Staurozoa, Cubozoa, Hydrozoa, and Scyphozoa) and both anthozoan classes (Octocorallia and Hexacorallia). Our results show that Hox genes involved in patterning the directive axes of anthozoan polyps are absent in the stem leading to Medusozoa. For the first time, we show spatial and temporal expression patterns of Hox and ParaHox genes in the upside-down jellyfish *Cassiopea xamachana* (Scyphozoa), which are consistent with diversification of medusozoan Hox genes both from anthozoans and within medusozoa. Despite unprecedented taxon sampling, our phylogenetic analyses, like previous studies, are characterized by a lack of clear homology between most cnidarian and bilaterian Hox and Hox-related genes. Unlike previous studies, we propose the hypothesis that the cnidarian–bilaterian ancestor possessed a remarkably large Hox complement and that extensive loss of Hox genes was experienced by both cnidarian and bilaterian lineages.

**Significance**

While bilaterian and cnidarian Hox genes both function in patterning the body axis, the evolutionary relationships between individual bilaterian and cnidarian Hox genes remain unknown. Despite applying the broadest cnidarian taxonomic sampling published to date to the problem, support for relationships between cnidarian and bilaterian Hox genes remains weak. Here, we point out the tendency for this weak support to be attributed to fast evolutionary change and instead propose that the lack of support is due to substantial gene loss in both the cnidarian and bilaterian Hox gene complements. This new outlook opens up the possibility for new ideas about the early evolution of animals and animal form.

## Introduction

More than 520 million years ago (McFadden et al. 2021), an animal existed that would give rise to both Cnidaria (a group that includes anemones, corals, and jellyfish) and Bilateria (a lineage that includes arthropods, mollusks, annelids, echinoderms, vertebrates, and the vast majority of other extant animal species). Bilateria is generally acknowledged to contain significant morphological diversity, but morphological descriptions of Cnidaria tend to focus on similarities within the group (i.e., gelatinous composition, tentacles, and specialized stinging cells). Nonetheless, there is extensive morphological diversity within Cnidaria (Daly et al. 2007) and investigating the evolutionary history of genes involved in specifying cnidarian body plans holds immense promise in understanding the evolution of this diversity.

The fundamental elements of the bilaterian body plan include an anterior–posterior axis, an orthogonal dorsal–ventral axis, and a through gut (with a few exceptions; e.g., the lack of through gut in Xenacoelomorpha, Jondelius et al. 2011). Within this shared body framework, a wide variety of morphologies have evolved. Similarly, cnidarians share a basic body plan: a single oral opening into a multifunctional body cavity defines the oral–aboral primary body axis. The cnidarian body plan takes two major shapes: the polyp and the medusa. In the polyp body form, the aboral end is generally attached to, or in contact with, a substrate, while the medusa body form (familiarly known as a “jellyfish”) is a free-swimming pelagic life history stage.

These body forms characterize the three clades within Cnidaria, the anthozoans, medusozoans, and endocnidozoans. The medusozoan life cycle includes both an asexually reproducing polyp stage and a sexually reproducing medusa stage, while the anthozoans form only polyps, which can reproduce both asexually and sexually. Endocnidozoans, consisting of the Myxozoa and the Polypodiozoa, are sister to medusozoans and display highly derived body forms specialized to their obligate parasitic life styles (Chang et al. 2015). In both anthozoans and medusozoans, sexual reproduction produces a planula stage, a swimming ciliated ovoid larva that develops immediately following embryogenesis and gives rise to the polyp. Distinctive morphological features distinguish the polyps and medusae of each class, so a thorough analysis of body form evolution and the genes involved requires representatives from each class. In particular, there are pronounced morphological distinctions between medusozoan and anthozoan polyps (Khalturin et al. 2019). Notably, some anthozoans (e.g., *Nematostella vectensis*) show bilateral symmetry that is defined primarily by mesenteries and a ciliated groove called the siphonoglyph that runs from the mouth into the pharynx (Hyman 1940). This bilateral symmetry defines a secondary body axis known as the directive axis, which is perpendicular to the oral–aboral axis.

Hox and ParaHox genes are homeobox-containing transcription factors present in both cnidarians and bilaterians, which have been implicated as key factors in the evolution of animal forms through their role in laying out the basic organization of body axes during embryonic development (Slack et al. 1993). By comparing the complement and developmental roles of Hox and ParaHox genes of cnidarians and bilaterians, it is possible to make inferences on how the evolution of these genes has influenced the morphological diversity between and within Cnidaria and Bilateria.

Hox and ParaHox genes have been investigated in detail in a range of bilaterian animals, so the evolution of these genes within Bilateria is relatively well understood (Lemons 2006). Far less is known about the evolution of these genes in cnidarians. Previous work identified Hox genes in a number of cnidarian species, with a focus on medusozoan classes Hydrozoa and Scyphozoa and anthozoan class Hexacorallia (Table 1). Understanding the phylogenetic relationships between cnidarian and bilaterian Hox and ParaHox genes will provide insight into the evolution of animal body plans, but these relationships remain unclear. Previous phylogenetic analyses have recovered weak support for relationships among these genes, and different analyses have disagreed about the relationships among these genes (Chourrout et al. 2006; Kamm et al. 2006; Ryan et al. 2007; Chiori et al. 2009; Dubuc et al. 2012). Increased taxon sampling has been shown to correlate with higher phylogenetic accuracy (Zwickl and Hillis 2002), so researchers have suggested cnidarian and bilaterian Hox gene relationships would resolve when phylogenies included enough cnidarian species from sufficiently diverse taxa. Indeed, no previously published phylogenies have included complete sets of cnidarian Hox and ParaHox genes from representatives of six major cnidarian lineages, meaning that increased taxon sampling is a critical next step in resolving these phylogenies.

**Table 1:** Studies With Phylogenetic Analyses of Cnidarian Hox Genes

Species	Class	Reference
*Podocoryna carnea*	Hydrozoa	(Aerne et al. 1995; Yanze et al. 2001)
*Eleutheria dichotoma*	Hydrozoa	(Kuhn et al. 1996; Kamm et al. 2006; Jakob and Schierwater 2007)
*Hydra viridissima*	Hydrozoa	(Gauchat et al. 2000)
*Hydractinia symbiolongicarpus*	Hydrozoa	(Cartwright et al. 2006)
*Hydra vulgaris* (formerly *Hydra magnipapillata*)	Hydrozoa	(Chourrout et al. 2006)
*Clytia hemisphaerica*	Hydrozoa	(Chiori et al. 2009)
*Turritopsis dohrnii*	Hydrozoa	(Quiquand et al. 2009)
*Nematostella vectensis*	Hexacorallia	(Finnerty and Martindale 1999; Chourrout et al. 2006; Ryan et al. 2006, 2007)
*Acropora digitifera*	Hexacorallia	(Dubuc et al. 2012)
*Cassiopea xamachana*	Scyphozoa	(Kuhn et al. 1999)
*Aurelia coerulea*	Scyphozoa	(Gold et al. 2019; Khalturin et al. 2019)
*Nemopilema nomurai*	Scyphozoa	(Kim et al. 2019)
*Rhopilema esculentum*	Scyphozoa	(Nong et al. 2020)
*Sanderia malayensis*	Scyphozoa	(Nong et al. 2020)
*Dendronephtya gigantea*	Octocorallia	(Jeon et al. 2019)
*Morbakka virulenta*	Cubozoa	(Khalturin et al. 2019)
*Polypodium hydriforme*	Polypodiozoa	(Chang et al. 2015)

Previous work has identified Hox genes in a number of cnidarians, but most of these studies have been limited to the medusozoan classes Hydrozoa and Scyphozoa and anthozoan class Hexacorallia. Hox genes have been identified in one cubozoan species and in one octocorallian species. No previously published Hox gene investigations have included species from Staurozoa.

We take advantage of the recent surge of publicly available cnidarian genome and transcriptome data (Table 2) to construct the first-published phylogeny to include sets of Hox and ParaHox genes from representatives of six major cnidarian lineages (Staurozoa, Cubozoa, Hydrozoa, Scyphozoa, Octocorallia, and Hexacorallia) and bilaterian representatives from Deuterostomia, Spiralia, and Ecdysozoa. We use the resulting tree to identify the previously undescribed Hox and ParaHox genes of staurozoans *Haliclystus sanjuanensis* and *Calvadosia cruxmellitensis;* cubozoan *Alatina alata*; hydrozoan *Craspedacusta sowerbii*; scyphozoan *Cas. xamachana*, octocorallians *Corallium rubrum, Eunicella cavolini, and Renilla reniformis*; hexacorallians *Anthopleura elegantissima* and *Lobactis scutaria*; and ceriantharian *Ceriantheopsis americana*. We then analyze the spatial and temporal expression of these genes in *Cas. xamachana* during embryonic development.

**Table 2:** Data Sources for Phylogenetic Analyses

Organism	Data	Data source/citation
*Homo sapiens*	Hox/ParaHox set	HomeoDB (Zhong et al. 2008)
*Branchiostoma floridae*	Hox/ParaHox set	HomeoDB (Zhong et al. 2008)
*Drosophila melanogaster*	Hox/ParaHox set	HomeoDB (Zhong et al. 2008)
*Tribolium castaneum*	Hox/ParaHox set	HomeoDB (Zhong et al. 2008)
*Capitella teleta*	Hox/ParaHox set	(Paps et al. 2015; Zwarycz et al. 2015)
*Crassotrea gigas*	Hox/ParaHox set	(Zwarycz et al. 2015; Zhang et al. 2012)
*Nematostella vectensis*	Hox/ParaHox set	(Dubuc et al. 2012)
*Acropora digitifera*	Hox/ParaHox set	(Dubuc et al. 2012)
*Hydra vulgaris*	Hox/ParaHox set	(Chiori et al. 2009)
*Clytia hemisphaerica*	Hox/ParaHox set	(Leclère et al. 2019)
*Eleutheria dichotoma*	Hox/ParaHox set	(Kamm et al. 2006)
*Podocoryna carnea*	Hox/ParaHox set	(Yanze et al. 2001)
*Alatina alata*	Transcriptome	(Ohdera et al. 2019)
*Anthopleura elegantissima*	Transcriptome	(Kayal et al. 2018)
*Calvadosia cruxmellitensis*	Transcriptome	(Kayal et al. 2018; Ohdera et al. 2019)
*Cassiopea xamachana*	Transcriptome and Genome	(Ohdera et al. 2019; Kayal et al. 2018)
*Craspedacusta sowerbii*	Transcriptome	(Zapata et al. 2015)
*Corallium rubrum*	Transcriptome	(Kayal et al. 2018)
*Eunicella cavolini*	Transcriptome	(Kayal et al. 2018)
*Haliclystus sanjuanensis*	Transcriptome	(Kayal et al. 2018)
*Lobactis scutaria*	Transcriptome	(Kayal et al. 2018)
*Renilla reniformis*	Transcriptome	(Kayal et al. 2018)
*Ceriantheopsis americana*	Transcriptome	doi:10.6071/M3K39S

Our phylogenetic analyses included previously published Hox gene sequences in addition to Hox and Hox-related sequences we identified in cnidarian transcriptomes.

## Results

We defined gene families based on the following criteria: the clade had to have a bootstrap support value of at least 50, and the clade had to be present in both the maximum-likelihood and both Bayesian trees. A bootstrap support value of 50 is lower than the typical standard for defining a clade, but it is higher than the bootstrap values for these clades recovered in previous studies of cnidarian Hox genes (Chourrout et al. 2006; Kamm et al. 2006; Ryan et al. 2007; Chiori et al. 2009; Dubuc et al. 2012), and the fact that we recovered these clades by both maximum-likelihood and Bayesian analysis further supports their validity. Newly identified sequences were named based on the earliest published medusozoan or anthozoan gene within the clade. For a more detailed explanation of nomenclature, see Materials and Methods.

Previously identified Hox genes fell into clades that contained only bilaterian, anthozoan, or medusozoan genes, rather than genes from a combination of those groups, with the exception of the clade that included both anthozoan Anthox6 and the medusozoan gene we have named Cnox1. In the case of non-Hox homeobox genes, clades included both cnidarian and bilaterian genes. The relationships among these moderately supported gene clades show bootstrap support below 50 and vary greatly among the maximum-likelihood and Bayesian trees, indicating evolutionary relationships between gene clades are inconclusive.

### Non-Hox Homeobox Genes

**Fig. 1.**
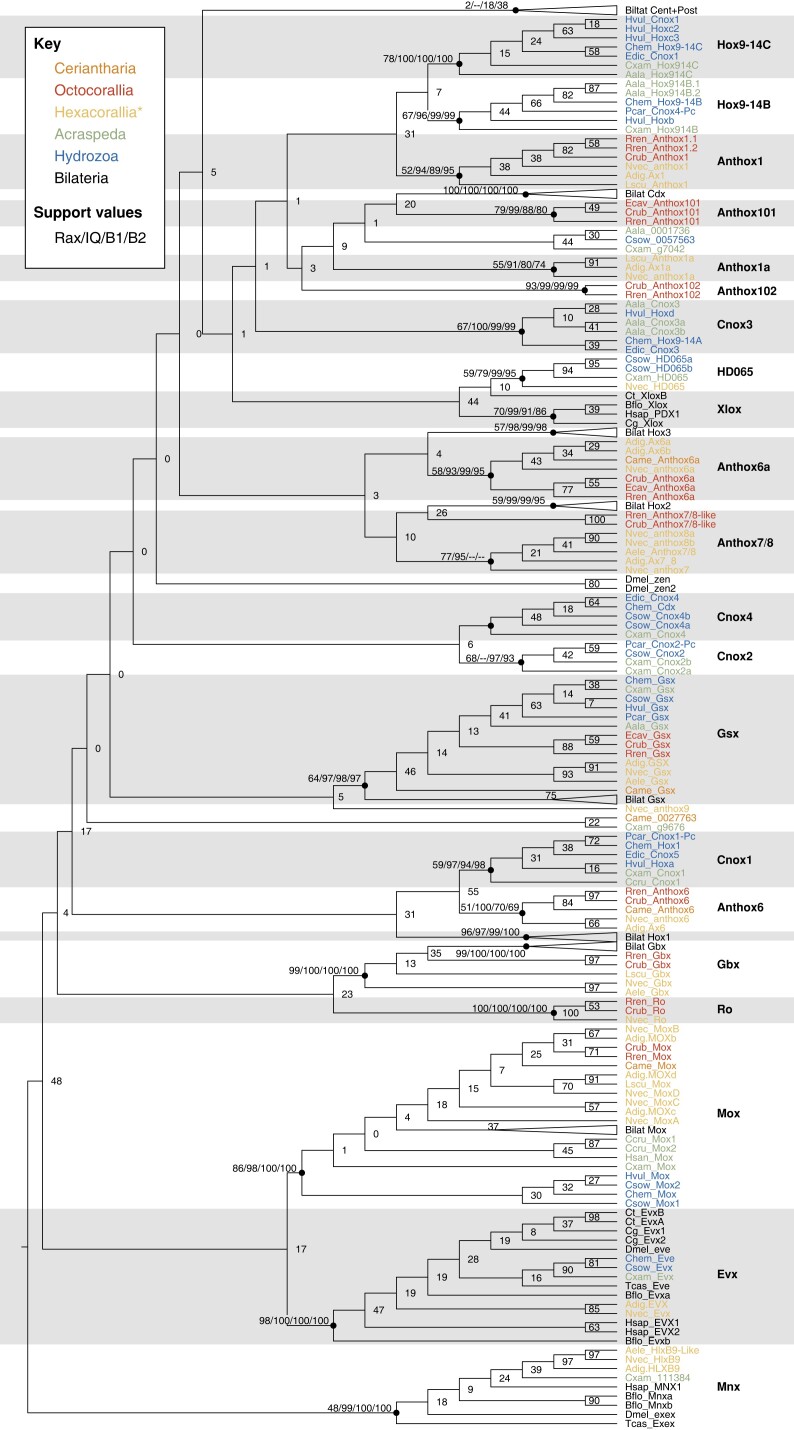
Maximum-likelihood phylogeny of cnidarian and bilaterian Hox and Hox-related genes. This tree was generated using RAxML with 25 maximum parsimony starting trees. The tree is rooted using the HlxB9/Mnx clade for display. Newly identified cnidarian homeodomains were named according to the oldest-published cnidarian gene in their clade. For each gene-defining clade, as well as for other important clades, we have included the bootstrap value for the RAxML tree, the bootstrap value for the IQ-TREE, and posterior probability values for both Bayesian trees (supplementary fig. S1, Supplementary Material online). While these gene clades show moderate support and are consistent across these differing analysis methods, the relationships among the clades are weakly supported and vary depending on the analysis method. Previous publications suggested these weakly supported relationships would become clearer with increased taxon sampling, but our analysis contains the broadest taxon sampling to date and still recovers very weak support for relationships among cnidarian and bilaterian Hox genes. Bilaterian taxa are in black (prefixes = Cg, Ct, Bflo, Dmel, Hsap, Tcas); octocorallian taxa are in red (prefixes = Crub, Ecav, Rren); non-ceriatnhed hexacorallian sequences are in yellow (prefixes=Nvec, Adig, Aele, Lscu); ceriantharian sequences are in orange (prefix = Came); hydrozoan sequences are in blue (prefixes = Chem, Csow, Edic, Hvul, Pcar); acraspedan (non-hydrozoan medusozoan) sequences are in green (prefixes = Aala, Ccru, Cxam, Hsan).

In our results, the clades that include both cnidarian and bilaterian genes were those for non-Hox family homeobox genes, and these clades generally showed higher bootstrap support than Hox gene clades (fig. 1). This strong support for gene clades containing bilaterian, anthozoan, and medusozoan genes indicated these genes were present in the last shared common ancestor of bilaterians and cnidarians and have been maintained since these two lineages split. These non-Hox clades included previously identified cnidarian and bilaterian homeobox genes as well as newly identified sequences from cnidarian transcriptomes. The best supported of these clades was Gbx, with bootstrap support of 99, including bilaterian genes as well as *N. vectensis* Gbx and newly identified sequences from anthozoans *R. reniformis, Co. rubrum, L. scutaria,* and *An. elegantissima*. Gbx included bilaterian and anthozoan genes but no medusozoan genes, concurring with Leclère and colleagues' finding that Gbx was lost in the medusozoan lineage (Leclère et al. 2019).

Ro is another clade which included anthozoan and bilaterian genes, but we mistakenly left bilaterian Ro genes out of our analysis. Supplementary figure S2, Supplementary Material online shows a reanalysis in which we added Ro genes from *Branchiostoma floridae, D. melanogaster,* and *Tribolium castaneum* to the alignment. These genes form a clade with *N. vectensis* Ro and newly identified sequences from *R. reniformis* and *Co. rubrum* (BS = 76).

Other non-Hox homeobox genes formed clades including bilaterian, anthozoan, and medusozoan genes. Evx formed a well-supported clade (BS = 98), including bilaterian genes; Evx genes from cnidarians *Acropora digitifera, Clytia hemisphaerica,* and *N. vectensis*; and newly identified sequences from cnidarians *Cr. sowerbii* and *Cas. xamachana* (fig. 1). Mox was another well-supported non-Hox clade (BS = 86), including Mox genes from bilaterians and from cnidarians *N. vectensis, Ac. digitifera*, *Ho. vulgaris,* and *Cl. hemisphaerica*, and newly identified sequences from *Co. rubrum, R. reniformis, Ce. americana, L. scutaria, Cal. cruxmellitensis,* and *Cr. sowerbii* (fig. 1). The ParaHox Gsx clade (BS = 64) included bilaterian Gsx genes; Gsx genes from *Cl. hemisphaerica, Ho. vulgaris, Podocoryna carnea, Ac. digitifera,* and *N. vectensis;* and newly identified sequences from *Cas. xamachana, Cr. sowerbii, Al. alata, Eu. cavolini, Co. rubrum, R. reniformis, An. elegantissima,* and *Ce. americana*.

NvHD065 formed a clade with a sequence from *Cas. xamachana* and two sequences from *Cr. sowerbii* (BS = 59). HD065 fell within a larger clade including bilaterian Xlox genes, but low bootstrap support for this clade (BS = 44) means the evolutionary relationship is less clear.

Similarly, *N. vectensis* and *Ac. digitifera* HlxB9 formed a well-supported clade with a new *An. elegantissima* sequence (BS = 97), which fell within a larger clade including bilaterian Mnx/Exex (formerly referred to as HlxB9) and a *Cas. xamachana* gene (BS = 48). Weak support for this clade as a whole makes the evolutionary scenario inconclusive.

### Relationships Between Cnidarian and Bilaterian Hox Genes

As in previously published analyses, our analyses showed strong support uniting some cnidarian and bilaterian Hox-related families (e.g., Gbx, Evx, Mox; fig. 1), but poor support for the majority of bilaterian- and cnidarian-uniting clades. In many cases, cnidarian genes formed clades with moderate support and bilaterian genes formed clades with moderate support, but in all cases, the bootstrap support uniting cnidarian and bilaterian Hox clades was below 50 and the pairings were often not replicated in the Bayesian trees. In cases where a bilaterian clade was sister to a cnidarian clade, the cnidarian clade consisted of only anthozoans or of anthozoans and medusozoans, never medusozoans only.

The one case where a bilaterian Hox gene was sister to a clade containing both anthozoan and medusozoan genes was Hox1 (fig. 1). Bilaterian Hox1 (BS = 96) was sister to a clade consisting of one medusozoan and one anthozoan clade. The anthozoan clade (Anthox6; BS = 51) contained Anthox6 from *Ac. digitifera* and *N. vectensis*, and sequences from *Ce. americana, Co. rubrum,* and *R. reniformis*. The medusozoan clade (Cnox1; BS = 59) contained *Cl. hemisphaerica* Hox1, *Eleutheria dichotoma* Cnox5, *Hydra vulgaris* Hoxa, *P. carnea* Cnox1-Pc, and sequences from *Cal. cruxmellitensis* and *Cas. xamachana*. These two cnidarian clades were sister groups at a bootstrap value of 55, but this combined cnidarian clade's sister relationship to Hox1 was weakly supported (BS = 31).

There were three weakly supported Hox/ParaHox clades consisting of bilaterian and anthozoan sequences in the maximum-likelihood tree. Bilaterian Cdx (BS = 100) was sister to a previously undescribed octocorallian clade containing sequences from *Co. rubrum, Eu. cavolini,* and *R. reniformis* (Anthox101; BS = 79), but this sister-group relationship was poorly supported (BS = 20). The anthozoan Anthox6a clade (*Ac. digitifera* Anthox6a and 6b, *N. vectensis* Anthox6a, *Ce. americana*, *Co. rubrum, Eu. cavolini,* and *R. reniformis*) was sister to the bilaterian Hox3 clade (BS = 4). Bilaterian Hox2 was sister to a pair of octocorallian sequences (*Co. rubrum* and *R. reniformis*), and this clade was sister to hexacorallian Anthox7/8 (*Ac. digitifera* Anthox7/8; *N. vectensis* Anthox7, 8a, and 8b, and a sequence from *An. elegantissima*; BS = 10). Weak support for these three clades casts doubt on whether these relationships are accurate.

The above relationships between cnidarian and bilaterian Hox/ParaHox genes are weakly supported, but the relationships between the remainder of cnidarian and bilaterian Hox/ParaHox genes are even less clear. The weakly supported clade uniting cnidarian HD065 and bilaterian Xlox (BS = 44) was sister to a large, extremely weakly supported clade (BS = 1) containing a number of moderately supported cnidarian clades and the bilaterian clade Cdx described below. One of these clades was the Cnox3 medusozoan clade (BS = 67) which contained *Cl. hemisphaerica* Hox9-14A, *El. dichotoma* Cnox3, *Hy. vulgaris* Hoxd, a sequence from *Al. alata,* and two sequences from *Cr. sowerbii*. The anthozoan Anthox1 clade (BS = 52) was sister to a pair of medusozoan clades. Anthox1 (BS = 52) included the titular gene from *Ac. digitifera* and *N. vectensis*, plus sequences from *Co. rubrum* and *L. scutaria* and two from *R. reniformis*. One medusozoan clade (Hox9-14B; BS = 67) contained *Cl. hemisphaerica* Hox9-14B, *Hy. vulgaris* Hoxb, *P. carnea* Cnox4-Pc, one sequence from *Cas. xamachana*, and two sequences from *Al. alata*. The other medusozoan clade (Hox9-14C; BS = 78) included *Cl. hemisphaerica* Hox9-14C; *El. dichotoma* Cnox1; *Hy. vulgaris* Cnox1, Hoxc2, and Hoxc3; one sequence from *Al. alata,* and one sequence from *Cas. xamachana*. The node connecting these two clades had a bootstrap value of 7, and the node connecting them to Anthox1 had a bootstrap value of 31. In the Bayesian trees, Anthox1 was sister to one medusozoan clade, with the other sister to both. Each of the three clades showed a sufficient bootstrap support to suggest that the genes within likely share a common ancestral gene, but poor support for relationships among the three clades means we cannot be confident about the relationships among the three genes.

Several small anthozoan clades were present on their own. A previously undescribed octocorallian-specific clade consisted of sequences from *Co. rubrum* and *R. reniformis* (Anthox102; BS = 93). Anthox1a appeared to be hexacorallian specific and consisted of sequences from *Ac. digitifera* and *N. vectensis* along with a sequence from *L. scutaria* (BS = 55). A previously undescribed rhopaliophoran (Cubozoa and Scyphozoa) clade consisted of sequences from *Al. alata* and *Cr. sowerbii* and two *Cas. xamachana* sequences (BS = 44), but because this bootstrap support was so low, suggesting these genes do not in fact share an evolutionary origin, we declined to name this clade and instead kept the identifying numbers from the genome or transcriptome.

In summary, we identified several well-supported non-Hox/ParaHox clades consisting of both cnidarian and bilaterian clades. However, many of the relationships between cnidarian Hox gene clades and bilaterian Hox gene clades were so poorly supported that we cannot be confident these apparent sister groups derived from the same ancestral gene in the cnidarian–bilaterian ancestor. The bilaterian central and posterior Hox gene clade included no cnidarian genes, suggesting these are bilaterian innovations or were lost in the cnidarian lineage. We also identified several clades consisting of newly identified cnidarian genes with no clear relationships to other clades, which may be innovations specific to these cnidarian lineages.

### Hypothesis Testing

**Fig. 2.**
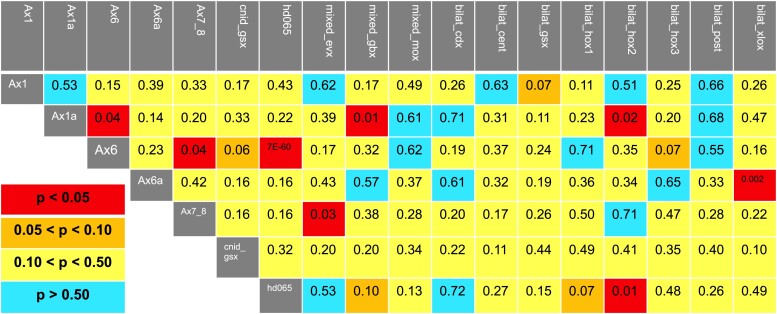
Hypothesis testing of potential sister-group relationships between cnidarian and bilaterian gene clades. We used AU tests (Shimodaira 2002) as implemented in IQ-TREE to test the likelihood of relationships between Hox and ParaHox gene families. We tested all possible pairs of cnidarian gene families as sister taxa (e.g., Anthox6 and Anthox6*a*). In addition, we tested all possible pairs of cnidarian and bilaterian families (e.g., Anthox6 and Hox1). Using the *P* < 0.05 criterion, we were able to reject only eight of our of 98 potential sister-group hypotheses. For cnidarian-specific clades, we rejected the sister-group hypotheses (Anthox6, Anthox1*a*), (Anthox6, Anthox7/8), and (Anthox6, HD065). For relationships between cnidarian-specific clades and clades containing both cnidarians and bilaterians, we rejected (Anthox1*a*, Gbx) and (Anthox7/8, Evx). For relationships between cnidarian-specific and bilaterian-specific clades, we rejected (Anthox1*a*, Hox2), (HD065, Hox2), and (Anthox6*a*, Xlox).

The relationships between bilaterian and cnidarian Hox and ParaHox genes were mostly weakly supported and, therefore, it was difficult to infer the evolutionary history of these genes. In an attempt to narrow down the possibilities of potential relationships, we tested potential sister relationships between these clades. Using the approximately unbiased (AU) test, a method for hypothesis testing maximum-likelihood tree topology (Shimodaira 2002), we tested 98 hypotheses and were only able to statistically reject eight of these (criteria for rejection was *P* < 0.05; fig. 2). We were able to reject the following cnidarian-specific clade hypotheses (Anthox6, Anthox1a), (Anthox6, Anthox7/8), and (Anthox6, HD065). Likewise, we were able to reject (Anthox1a, Hox2), (HD065, Hox2), and (Anthox6a, Xlox), which bring together cnidarian and bilaterian clades. We were also able to reject the hypotheses (Anthox1a, Gbx) and (Anthox7/8, Evx), which group cnidarian-specific clades with clades containing both cnidarian and bilaterian species.

In addition to the 98 sister-group relationships, we tested four hypotheses grouping more than two Hox gene clades into a larger clade, none of which could be rejected. These include: (Anthox1, Anthox1a, bilaterian posterior Hox genes, bilaterian central Hox genes; *P* = 0.32), (Anthox1, Anthox1a, bilaterian posterior Hox genes; *P* = 0.53), (Anthox6, Anthox6a, Hox1; *P* = 0.50), and (Cdx, Xlox, HD065; *P* = 0.50).

### *In Situ* Hybridization Expression Patterns

In the *Cas. xamachana* transcriptome and genome, we identified the ParaHox genes CxGsx and CxHD065; the Hox-related genes CxEvx, Cx111384, and CxMox; and the eight Hox genes CxHox9-14C, CxHox9-14B, CxCnox4, CxCnox2a, CxCnox2b, CxCnox1, Cx_g7042, and Cx_g9676. Four of these 13 showed expression at or before the planula stage of development by in situ hybridization, specifically in spatially restricted regions along the oral–aboral axis (CxHox9-14B, CxHox9-14C, CxCnox2a, and Cx_g9676). Of these four, three have been published in other species (CxHox9-14B, CxHox9-14C, and CxCnox). The gene Cx_g9676 did not group into a sufficiently well-supported clade for us to give it a name, so it is identified here by the sequence number in the genome.

**Fig. 3.**
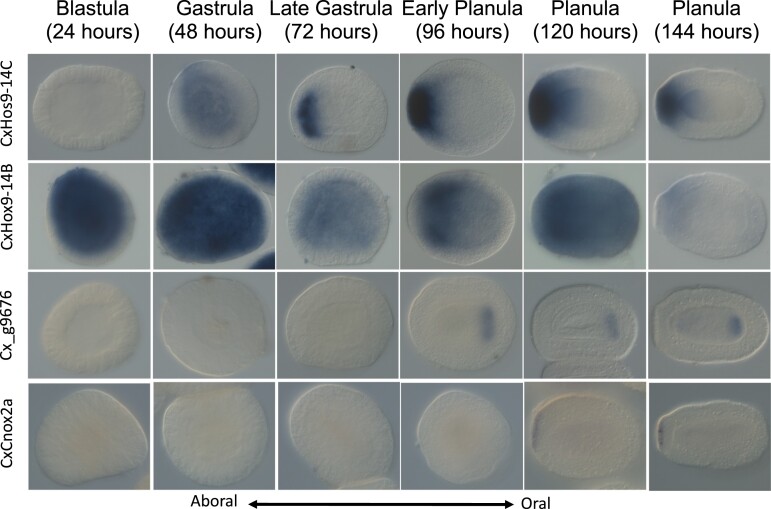
*In situ* expression patterns of *Cassiopea xamachana* Hox genes during early development. Of the 13 Hox, ParaHox, and Hox-like genes we identified in *Cas. xamachana*, four show visible expression patterns by in situ hybridization during embryonic development. Times listed are hours counted after first cleavage. All embryos are shown with the future oral pole facing to the right in stages where the oral and aboral poles can be distinguished. CxHox9-14*C* shows spatially generalized expression by 48 h after first cleavage, and by 72 h, expression is localized to the future aboral pole. Aboral expression continues through the 144-h time point. Aborally localized expression has been observed for NvAnthox1 in *N. vectensis* as well as ChemHox9-14*C* in *Clytia hemisphaerica*. CxHox9-14*B* shows spatially generalized expression by 24 h after first cleavage, which becomes localized to a ring around the aboral pole by 96 h. Cx_g9676 is a newly identified scyphozoan gene. It shows endodermal expression at the future oral pole starting at 96 h. CxCnox2*a* shows aborally localized expression starting at 120 h.

**Fig. 4.**
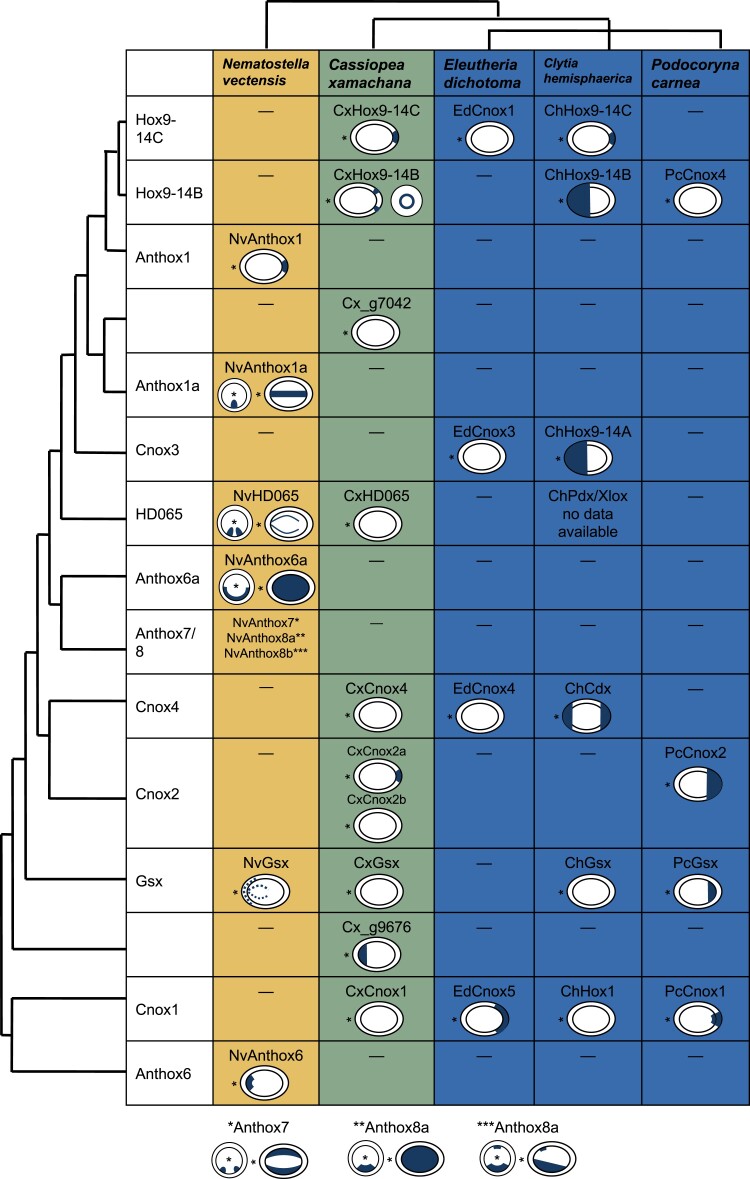
Comparison of Hox gene planula expression patterns across Cnidaria. Hox gene expression patterns show variation across Cnidaria. Hox expression patterns in *Cas. xamachana* are novel findings presented for the first time in this paper. Asterisks indicate the oral pole of the planula. Anthox1/Hox9-14*C* is notable for its consistent pattern of aborally localized expression in anthozoan *N. vectensis*, hydrozoan *Cl. hemisphaerica*, and medusozoan *Cas. xamachana*. One of the Cnox2 genes in *Cas. xamachana* shows aboral expression consistent with the aboral expression pattern published in *P. carnea*. Boxes with dashes indicate that a gene has not been identified in the species. *N. vectensis*, *Cas. xamachana*, and *Cl. hemisphaerica* have completed genomes, so genes that have not been identified are likely not present in the species. *Eleutheria dichotoma* and *P. carnea* have transcriptomes only, so it is possible genes are present even if they have not been identified.

CxHox9-14B was expressed throughout the embryo by 24 h after first cell division, and by 4 days, expression becomes restricted to an ectodermal ring around the aboral pole, excluding the pole itself (fig. 3). In comparison, *Cl. hemisphaerica* Hox9-14B is expressed in the oral half of the planula (Chiori et al. 2009), and *P. carnea* Cnox4 is expressed orally in the early embryo but not detected in the planula (Yanze et al. 2001; figure 4).

CxHox9-14C was expressed starting approximately 48 h after the first cell division (fig. 3). By 72 h, expression was restricted to the aboral region of the embryo, and this pattern was maintained through at least 6 days. The *N. vectensis* and *Cl. hemisphaerica* homologs of this gene show a similar aborally restricted pattern (Ryan et al. 2007; Chiori et al. 2009; figure 4).

CxCnox2a was expressed in the aboral ectoderm beginning 5 days post-fertilization (fig. 3). Masuda-Nakagawa et al. also reported aboral Cnox2 expression in the *P. carnea* planula (Masuda-Nakagawa et al. 2000) though this expression pattern covers a much larger area than what we observed in *Cas. xamachana* (fig. 4).

The *Cas. xamachana* gene numbered g9676 in the genome was part of no clades with a bootstrap value above 50, meaning it has not been identified in other species studied here and may be a scyphozoan novelty. It was expressed starting at 96 h in the oral region of the endoderm, and this expression pattern continued through the planula stage (fig. 3).

## Discussion

The vast majority of cnidarian Hox and ParaHox gene analyses have focused on species from Hexacorallia and Hydrozoa. Recent genome studies have examined and expanded the availability of the Hox and ParaHox gene complements of a number of species (Gold et al. 2019; Khalturin et al. 2019; Kim et al. 2019; Jeon et al. 2019). However, these studies have not included phylogenetic analyses of complete sets of Hox and ParaHox genes from a broad sampling of cnidarian classes. The phylogenetic analyses we present here represent the broadest taxon sampling to date of cnidarian Hox and ParaHox genes. In addition to characterizing the Hox and ParaHox genes of the scyphozoan *Cas. xamachana,* we have identified previously undescribed Hox and ParaHox genes from the cubozoan *Al. alata*; the staurozoans *Ha. sanjuanensis* and *Cal. cruxmellitensis*; the octocorals *R. reniformis* and *Co. rubrum*; and the ceriantharian *Ce. americana*. It should be noted that we have used transcriptome data from most of these species, and as such, there may be additional genes we have not identified here. Additionally, we did not include data from Endocnidozoa (i.e., Myxozoa and Polypodiozoa) in the main paper, but we have included an analysis of *Polypodium hydriforme* in the supplement (supplementary fig. S2, Supplementary Material online. Our results replicate previously observed weak support for relationships between cnidarian and bilaterian Hox genes, which we propose may be at least partially explained by extensive gene loss in both cnidarian and bilaterian Hox lineages.

### Gene Loss may Explain Relationships Between Cnidarian and Bilaterian Hox Genes

**Fig. 5.**
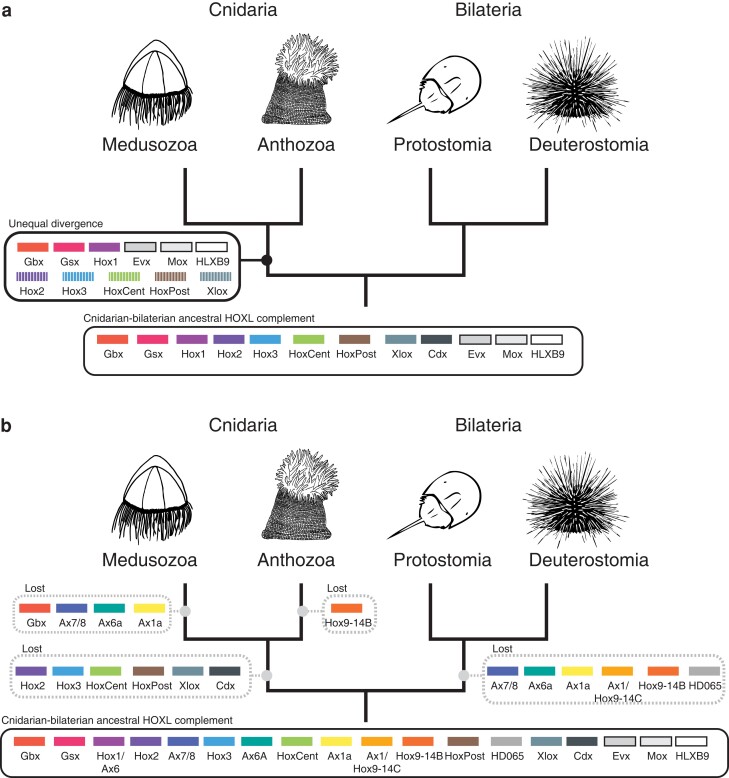
Hypothesis that cnidarian and bilaterian lineages have both lost a number of Hox and Hox-like genes since their last common ancestor. Historically, analyses of Hox genes between Bilateria and Cnidaria have tried to find common ancestral predecessors shared between the two clades. This form of analysis tends to prioritize the fitting cnidarian Hox/ParaHox/Hox-like genes into more well-known bilaterian Hox gene families. Figure 5*a* graphically represents this approach, indicating how cnidarian Hox and ParaHox genes have been shoehorned into bilaterian categories. In this paradigm, the major changes in Hox genes have occurred solely in the cnidarian lineage, and cnidarian Hox genes are simply bilaterian Hox genes modified beyond recognition, as represented by the striped boxes. We base our new hypothesis (depicted in fig. 5*b*) on the fact that Cnidaria and Bilateria have had equal time to diverge since their taxonomic split and the poor support consistently recovered for phylogenetic trees relating bilaterian Hox gene to cnidarian Hox genes. Instead of divergence from an ancestor with a limited Hox complement, we hypothesize that cnidarian and bilaterian Hox complements have evolved by notable loss of Hox genes from the ancestral state. We present these two scenarios not as binary answers to the question of Hox/ParaHox evolution, but as poles of a spectrum with the truth lying somewhere in between the two. Historically, hypotheses of Hox gene scenarios have fallen close to figure 5*a*, and we propose that phylogenetic results are consistent with a scenario that includes elements of figure 5*b*.

Previous studies attempting to establish orthology between extant cnidarian and bilaterian Hox genes have implicitly assumed the most recent shared ancestor of cnidarians and bilaterians had a complement of Hox genes similar to that of extant bilaterians (fig. 5*a*). When previous work recovered weak support for cnidarian and bilaterian Hox gene relationships, researchers suggested increased taxon sampling would strengthen support, making orthologous relationships clear (Finnerty and Martindale 1998; Ferrier and Holland 2001; Ferrier and Minguillón 2003; Dubuc et al. 2012). Instead, our broad taxon sampling still produced weakly supported relationships between cnidarian and bilaterian Hox genes, with these relationships varying depending on whether maximum-likelihood or Bayesian approaches were applied. If we assume the ancestral Hox complement was similar to bilaterians, our results, like the results of previous studies, would require that this divergence between lineages has been so great that cnidarian and bilaterian gene orthologues are now effectively unrecognizable.

It is possible that the weak support values are entirely the result of extreme divergence in the homeobox sequences, as previous work has implicitly assumed. However, we propose the relationship between cnidarian and bilaterian gene complements can also be explained by an evolutionary scenario in which gene loss played a larger role than previously considered (fig. 5*b*). Under this scenario, the number of Hox genes in the last common ancestor of cnidarians and bilaterians was considerably larger than the ten Hox genes and three ParaHox genes hypothesized to be present in the last common ancestor of flies and humans (Balavoine and de Rosa 2002). After the divergence of cnidarians and bilaterians, each lineage lost distinct sets of Hox/ParaHox genes. For example, the cnidarian–bilaterian ancestor may have had a gene that gave rise to the cnidarian Anthox6/Cnox1 clade, which was lost in bilaterians, and another gene that gave rise to Hox1, which was lost in cnidarians. In this scenario, the placement of Anthox6/Cnox1 and Hox1 as sister groups is the result of convergence or weak homology that predates the establishment of these two families, rather than strict orthology.

Figure 5 depicts the two poles of a spectrum that ranges from the “extreme divergence” paradigm (fig. 5*a*) to an “extreme gene loss” scenario (fig. 5*b*), with the true evolutionary trajectory likely somewhere between these two hypotheticals. Historically, studies of Hox and ParaHox genes have leaned heavily toward the “extreme divergence” scenario to the exclusion of even considering gene loss. Because figure 5 shows the most extreme version of each paradigm, it includes unlikely possibilities, such as the presence of medusozoan-specific gene Hox9-14B in the cnidarian–bilaterian ancestor and subsequent loss in both the bilaterian and anthozoan lineages. This particular evolutionary trajectory is unlikely, but so is a trajectory in which every single cnidarian Hox sequence has diverged so much from a bilaterian-like ancestral gene so as to be unrecognizable. We argue that both gene divergence and gene loss have likely played roles in the evolution of cnidarian and bilaterian Hox and ParaHox genes, but that gene loss has played a much more significant role than previously thought and should be seriously considered in future work.

The role of gene loss in evolution is not a new or controversial idea (reviewed by Albalat and Cañestro 2016). Recent work suggests much more gene loss has occurred in metazoan genome evolution than previously thought (Fernández and Gabaldón 2020; Guijarro-Clarke et al. 2020), and Hox gene loss has been observed in several lineages (Aboobaker and Blaxter 2003; Butts et al. 2010). Fernández and Gabaldón (2020) single out Cnidaria as a site of particularly drastic gene loss, estimating 15% of genes may have been lost in the cnidarian stem lineage. Evolution by gene loss is a well-documented phenomenon and is consistent with the pattern of low support values consistently seen in phylogenetic analyses of cnidarian and bilaterian Hox genes.

### Implications for the Evolution of the Anthozoan Directive Axis

Previous work on cnidarian Hox genes has provided conflicting evidence about the relationship between cnidarian and bilaterian body axes. Both the directive axis (Arendt 2018) and the oral–aboral (DuBuc et al. 2017) axis have been suggested to be homologous to the bilaterian anterior–posterior axis (reviewed by Technau and Genikhovich 2018).

*In situ* hybridization analyses have shown that six *N. vectensis* homeobox genes (Gbx, Anthox1a, Anthox6a, Anthox7, Anthox8a, and Anthox8b) are differentially expressed along the directive axis during development (Ryan et al. 2006). He et al. (2018) showed that knockdown of Gbx, Anthox1a, Anthox8a, or Anthox6a resulted in the loss of the region of the directive axis where they were expressed in *N. vectensis*. These genes are expressed in an overlapping pattern along the directive axis and control axial patterning in a manner some have compared with the posterior prevalence seen in bilaterian Hox genes, leading some to propose the directive axis is homologous to the anterior–posterior axis (Arendt 2018). However, the cnidarian Hox genes involved in patterning the directive axis (Anthox1a, Anthox8a, and Anthox6a) have no clear bilaterian homologs (fig. 1). Therefore, resemblance between the expression patterns of these cnidarian genes and the posterior prevalence pattern of bilaterian Hox genes provides no evidence for homology of the cnidarian directive axis and bilaterian anterior–posterior axis.

As with hydrozoan Hox genes (Leclère et al. 2019), we did not recover medusozoan orthologs of Hox genes involved in patterning the anthozoan directive axis (Anthox6, Anthox7, Anthox8), suggesting these genes were lost in the stem medusozoan or are an anthozoan innovation. This result extends to all medusozoans the hypothesis promoted by Leclère and colleagues (2019) that extensive genomic evolution (in this case, gene loss) has contributed to the simplification of the medusozoan polyp.

Given the lack of support for orthology between cnidarian and bilaterian Hox families, upstream regulators of Hox genes may be more informative than Hox genes for determine axial homology between Cnidaria and Bilateria. For example, the BMP/Chordin system provides evidence for homology between the dorsal–ventral and directive axis: opposing BMP and Chordin gradients pattern the dorsal–ventral axis in diverse bilaterians (Holley et al. 1995; de Robertis and Sasai 1996; Lowe et al. 2006; Tan et al. 2017; Akiyama-Oda and Oda 2006), and opposing NvBMP2/4 (also called NvDpp) and NvChordin gradients pattern the directive axis in *N. vectensis* (Finnerty et al. 2004; Rentzsch et al. 2006). Homology of bilaterian and anthozoan BMP and Chordin implies this gene system was present in the last common ancestor of cnidarians and bilaterians. In *N. vectensis*, knockdown of NvBMP2/4 or NvChordin prevents expression of NvAnthox1a and NvGbx at the planula stage (Saina et al. 2009), and NvBMP2/4 knockdown prevents expression of NvAnthox8 (Leclère and Rentzsch 2014). Additionally, based on strong support for the orthology of the bilaterian and anthozoan Gbx gene (BS = 99), it seems likely that Gbx was present in the cnidarian–bilaterian ancestor. Homology of the BMP/Chordin system and of Gbx between anthozoans and bilaterians supports homology of the anthozoan directive axis and the bilaterian dorsal–ventral axis. Our data suggest that Hox genes are not reliable markers for determining axial homology and that investigating secondary-axis patterning systems such as BMP/Chordin and others may be more informative.

### Implications for Body Form Evolution

BMP/Chordin and Gbx function in patterning bilateral symmetry in *N. vectensis* and were present in the cnidarian–bilaterian ancestor, suggesting the cnidarian–bilaterian ancestor could have been bilaterally symmetrical. In this scenario, radially symmetrical medusozoans evolved from a bilaterally symmetrical ancestor. Homology of anthozoan and bilaterian bilateral symmetry genes that are absent in medusozoans suggests medusozoans lost bilateral symmetry secondarily.

There is precedent for gene loss corresponding to morphological change. Babonis et al. (2018) found that genes involved in tentacle development were absent in a lineage of ctenophores that has lost their tentacles, and Espregueira Themudo et al. (2020) reviewed gene losses in cetaceans that correspond to cetacean skin's aquatic adaptations. Leclère et al. (2019) observed a number of gene losses in the hydrozoan *Cl. hemisphaerica* that correspond to secondarily simplified polyps and planulae, supporting a scenario in which gene loss drove morphological change in the medusozoan lineage. Development of the medusa life stage meant both a transition to a new environment and a reduction in functions performed by the polyp, both possible drivers of, or opportunities for, morphological change.

## Conclusion

We hypothesize that the cnidarian–bilaterian ancestor had a relatively large complement of Hox genes, and that extensive Hox gene loss occurred in both the cnidarian and bilaterian lineages. This hypothesis is consistent with our results and with previous work on cnidarian and bilaterian Hox and ParaHox genes. We come to this hypothesis based on a lack of support for homology between cnidarian and bilaterian Hox gene groups, even with extensive sampling and a variety of phylogenetic methodologies. Hypothesis testing allows us to reject a number of cnidarian–bilaterian gene sister-group relationships, but ultimately does not narrow down likely sister-group relationships enough to point to any likely homologies. Gene loss provides an elegant explanation for the absence of homologous Hox genes between cnidarians and bilaterians. Other work provides evidence that gene loss played a role in evolution of major metazoan lineages (Fernández and Gabaldón 2020; Guijarro-Clarke et al. 2020). Therefore, it is reasonable to conclude that gene loss may have played a role in the evolution of cnidarian and bilaterian Hox and ParaHox genes. Furthermore, homology between a dorsal–ventral patterning mechanism and a directive axis patterning mechanism suggests the cnidarian–bilaterian ancestor was bilaterally symmetrical, and medusozoan radial symmetry may have evolved from the loss of bilateral symmetry patterning genes.

Future work examining expression patterns and function of the Hox and ParaHox genes we have identified in diverse cnidarians will provide further insight into the evolution of these genes, including conservation and divergence of function. Currently, Hox gene function across cnidaria is understood in only a few cases. Our analyses suggest the potential for new and exciting work on Hox genes in a broader-than-ever sampling of cnidarians.

## Materials and Methods

### Reproducibility and Transparency Statement

Phylotocol, custom scripts, command lines, and alignments used in these analyses, and trees resulting from these analyses, are available at https://github.com/josephryan/Steinworth_CnidarianHox. Release v1.0 of this repository, which represents the state of the repo at publication time, has been deposited on Zenodo (doi: 10.5281/zenodo.7463438).

To maximize transparency and minimize confirmation bias, all phylogenetic analyses were pre-planned using a phylotocol (DeBiasse and Ryan 2019) posted to this GitHub site. We made three changes to our original plan during the life of this project, and these changes were documented and justified in the phylotocol. First, after originally removing all cnidarian homeodomain sequences containing more than five gaps, we realized that it was important to include all potential *Cas. xamachana* Hox/ParaHox homeodomains, and so returned to our alignment those *Cas. xamachana* homeodomains that had been removed. For those with five or more gaps (CxCnox2a, CxCnox4, and CxHD065), we replaced the initial gap-containing sequences with the complete sequences we cloned. Second, we performed an additional pruning step, which described in detail in the phylogenetic analyses section below. The third change we made to the Phylotocol was in the AU test step. Initially, we planned to only perform the AU test on a few select potential sister-group pairings. When we found that very few of our hypotheses were rejected in this analysis, we decided to perform the AU test for the potential sister-group relationship between every possible combination of cnidarian clade plus cnidarian or bilaterian clade.

### Compiling Homeodomain Dataset

We compiled Hox/ParaHox homeodomains based on previous curated datasets from five bilaterians and six cnidarians (species and sources listed in Table 2). In addition, we identified homeodomain-containing genes from cnidarian transcriptomes (species and sources listed in Table 2) by conducting HMMer (version 3.1b2, Finn et al. 2011) searches with the hd60.hmm hidden Markov model from Zwarycz et al. (2015). We aligned homedomains directly to the hidden Markov model during the search using the hmm2aln.pl program (available in GitHub repository, supplementary File S1, Supplementary Material online), which utilizes this capability in the hmmsearch tool. From the resulting alignment, we removed non-Hox/ParaHox genes by generating a maximum-likelihood tree with IQ-TREE version 1.6.10 (Nguyen et al. 2015) and pruning those sequences that fell outside the smallest possible clade that encompassed all of the *N. vectensis* Hox/ParaHox genes (the custom Perl script for this step, make_subalignment2, is available in the GitHub repository). We cloned all *Cas. xamachana* genes that fell within this clade (see below for cloning methods).

To generate the dataset for the trees shown in this paper, we went back to the pre-pruned dataset and removed all homeodomain sequences containing five or more gaps, replacing the *Cas. xamachana* sequences that included gaps with the ones we cloned and sequenced. Then we again pruned non-Hox/ParaHox genes as described above. Cloning *Cas. xamachana* genes with gaps allowed us to include *Cas. xamachana* genes that otherwise would have been excluded. It is therefore likely that we have missed some genes from other species, but we still chose to remove sequences with five or more missing amino acids to ensure that our results were based on high-quality data.

Because the resulting tree made from this subalignment appeared to still contain sequences outside the clade defined by *N. vectensis* Hox/ParaHox genes, we repeated the pruning step and used the resulting alignment for further analyses. This additional pruning step was a deviation from our Phylotocol.

### Phylogenetic Analyses

We used a range of strategies to generate a tree with the highest likelihood given the data. We ran RAxML version 8.2.12 (Stamatakis 2014) with 25 starting parsimony trees and with 25 random starting trees, as well as one IQ-TREE, which includes 100 starting parsimony trees by default (Nguyen et al. 2015).

Maximum-likelihood trees used the GAMMA model of rate heterogeneity and LG amino acid substitution model, and rapid bootstrapping. Two independent runs of MrBayes version 3.2.7a (Ronquist et al. 2012) did not converge after 10,000,000 generations, so we generated a strict consensus tree for each of the two runs (supplementary fig. S1, Supplementary Material online). The Bayesian analysis used the Jones amino acid model, which was selected by the MrBayes program's fixed-rate model estimation.

We calculated likelihood scores for all analyses (including our Bayesian trees) using RAxML. We selected the tree with the highest likelihood as our primary phylogeny. All other trees are provided on our GitHub repository, which is also available at Zenodo (doi: 10.5281/zenodo.7463438).

When multiple isoforms with identical homeobox sequences were observed, we pruned the duplicate sequence, specifically in the cases of Csow_Evx, Cxam_Cnox1, and Cxam_111384. For *Cas. xamachana*, when identical copies of a sequence were observed from the genome and transcriptome, we pruned the transcriptome sequences, specifically in the cases of Cxam_g9676 and Cxam_g7042.

### Hypothesis Testing

We used AU tests (Shimodaira 2002) as implemented in IQ-TREE to test the likelihood of many plausible relationships of Hox and ParaHox gene families. We tested all possible pairs of cnidarian gene families as sister taxa (e.g., Anthox6 and Anthox6a). In addition, we tested all possible pairs of cnidarian and bilaterian families (e.g., Anthox6 and Hox1). Lastly, we tested four additional groupings that had been hypothesized in previous studies: (Anthox1, Anthox1a, bilaterian posterior Hox genes, bilaterian central Hox genes), (Anthox1, Anthox1a, bilaterian posterior Hox genes), (Anthox6, Anthox6a, Hox1), and (Cdx, Xlox, HD065).

### Gene Nomenclature

There is notable historically driven variation across cnidarian Hox gene names within the same clade, which makes naming new members of each gene family challenging. Currently, there is no standard naming convention for cnidarian Hox genes. In the absence of a system such as that implemented in vertebrate Hox genes (Scott 1992), we have decided on the following conventions for the purposes of this paper.

We have named newly identified Hox genes based on the earliest published medusozoan or anthozoan gene name in their clade, keeping medusozoan and anthozoan clades separate. For example, the medusozoan gene Cnox1 is so named because the *P. carnea* gene Cnox1 is the earliest published member of that clade. Therefore, we gave the name Cnox1 to the previously unnamed homeodomains in this clade from *Cal. cruxmellitensis* and *Cas. xamachana*. The clade also contains *Hy. vulgaris* Hoxa, *El. dichotoma* Cnox5, and *Cl. hemisphaerica* Hox1; we refrained from changing previously published names to minimize confusion. In the sister anthozoan clade, the earliest published gene name was Anthox6 in *N. vectensis* and *Ac. digitifera*, so newly identified sequences in *R. reniformis, Co. rubrum*, and *Ce. americana* were named Anthox6. *Podocoryna carnea* Cnox1 was published before the anthozoan Anthox6 genes, but we kept anthozoan and medusozoan gene names separate due to divergent and strongly established naming traditions in each lineage. Indeed, the age of the last common ancestor of Medusozoa and Anthozoa (∼571 Myr according to TimeTree 5) compared with the last common ancestor of Cnidaria and Bilateria (∼685 Myr according to TimeTree 5) is comparable (Kumar et al. 2017).

There were two exceptions to this “first-published” rule necessary to ensure every gene had a unique name. First, if the earliest published gene name in the clade conflicts with a name already used for another clade, we used the next oldest name. For example, *P. carnea* Cnox4 was published before *Cl. hemisphaerica* Hox9-14B, but *El. dichotoma* Cnox4, the titular gene of a separate clade, was published before either gene. Therefore, previously unnamed genes in the clade containing *P. carnea* Cnox4 and *Cl. hemisphaerica* Hox9-14B were named Hox9-14B.

The second exception to our “oldest-published” rule was that we refrained from using names in which the word “Hox” was followed immediately by the letter A, B, C, or D. This was done to prevent cnidarian Hox genes from sharing a name with the commonly used names of the vertebrate Hox clusters: HoxA, HoxB, HoxC, and HoxD. An example where all three of our rules applied was Hox9-14C: the earliest published gene in this clade is *El. dichotoma* Cnox1, but *P. carnea* Cnox1 was published earlier and we therefore used it to title a different clade. Next published was the *Hy. vulgaris* gene Cnox1, which suffers from the same problem in that it was already used to define a different clade, and Hoxc2, in which the letter “C” immediately follows “Hox.” Therefore, we named newly identified genes in this clade for the *Cl. hemisphaerica* Hox9-14C.

In two cases, newly identified anthozoan sequences formed clades containing no previously published genes. We have named these genes Anthox101 and Anthox102, arbitrarily selecting these numbers to follow the Anthox gene naming pattern but to be discontinuous with other numbered Anthox genes to avoid making false implications about genome positioning.

When newly identified sequences did not group into any clades, we did not name the gene and instead used the identification number from the genome or transcriptome.

### Collection of Biological Material From *Cassiopea xamachana*

Adult *Cas. xamachana* medusae were collected from Key Largo during May 2018 and kept in flow-through seawater tanks on a 12–12 h light cycle at the Whitney Laboratory for Marine Bioscience in St. Augustine, FL. Male and female medusae were kept together, and female medusae produced brooded zygotes daily approximately two hours after the lights turned on in the morning. Embryos can be dislodged from the female medusa's brooding appendages by applying a stream of water using a pipette. Zygotes underwent their first cell division approximately 2 h after they were first observed in the female medusa's brooding region. Because we are unable to pinpoint the moment of fertilization, embryo ages are counted from time of first cell division. At room temperature (approximately 25 °C) embryos reach the blastula stage by 24 h and the gastrula stage by 48 h. Gastrulae continue to elongate until reaching a planula stage at the age of 6 days, after which point further significant morphological change is not observed until planulae encounter a settlement cue.

### Cloning Genes From *Cassiopea*

Primers used to clone genes are included in supplementary Table S1, Supplementary Material online. We extracted RNA from *Cas. xamachana* zygotes, embryos, planulae, polyps, medusae, and ovaries using David A. Gold's protocol (supplementary File S2, Supplementary Material online) and used Ambion RT-for-qPCR reverse transcription kit (Cat. # 639505) to synthesize cDNA from mixed RNA. We amplified *Cas. xamachana* Hox and ParaHox genes from our cDNA using New England Biolabs Taq polymerase (Cat. # M0273; see Supplementary material for primers), DNA was gel-purified using the QiaQuick Gel Extraction Kit (Cat. # 28704), and fragments were ligated into pGEM-T plasmid using the Promega pGEM-T Vector Systems kit (Cat. # A3600). Plasmid was transformed into DH5-alpha competent cells. After cells were plated, we selected colonies with the correct size fragment, grew them in Luria broth (LB), and isolated plasmid DNA using the ThermoFisher Scientific GeneJET Plasmid Miniprep Kit (Cat. # K0502) and sent for sequencing at Macrogen, Inc. to confirm the clone contained *Cassiopea* sequence. *In situ* hybridization probes were synthesized from amplified target sequence using the Clontech Advantage RT-for-PCR kit with Roche digoxigenin-11-UTP (Cat. # 3359247910). A detailed *in situ* hybridization protocol is available at doi:10.17504/protocols.io.biz4kf8w.

We were unable to amplify genes Cxam_Evx, Cxam_Mox, Cxam_Cnox2a, Cxam_Cnox2b, Cxam_Cnox1, and Cxam_111384 from cDNA. Instead, we ordered the sequences as gene blocks from Integrated DNA Technologies and used them to create in situ hybridization probes as described below; none of these probes produced expression patterns.

### In Situ Hybridization

Whole-mount in situ hybridization protocol is modified from (Sinigaglia et al. 2018; Wolenski et al. 2013).

Tissues were fixed for 1.5 min in 4% paraformaldehyde with 0.3% glutaraldehyde in phosphate-buffered saline with 1% Tween (PTw), then fixed for 1 h at 4 °C in 4% paraformaldehyde in PTw. Fixed tissues were stored in 100% methanol at −20 °C, then rehydrated into PTw before in situ hybridization.

Rehydrated tissues were treated with triethanolamine and acetic anhydride, then rinsed with hybridization buffer (4 M urea, saline-sodium citrate buffer at pH 4.5, 50 ug/ml heparin, 0.10% Tween, 1% sodium dodecyl sulfate) with 100 ug/ml salmon sperm. Tissues were left in hybridization buffer for at least 1 h at 63 °C before probes were applied. Probes were diluted to a concentration of 1 ug/ml and heated to 95 °C for 5 min before application to tissues. Tissue remained in probe solution overnight.

After probe removal, tissues were gradually transferred to 0.02 × saline-sodium citrate (SSC) buffer, then to PTw. Tissues were blocked for at least 1 h in Roche blocking buffer (diluted from 10 × to 1 × in maleic acid buffer), then incubated overnight at 4 °C in Roche blocking buffer with 1:5000 concentration of antidigoxigenin Fab fragments antibody. Antibody was washed off with phosphate-buffered saline containing 1% Triton. Probe was visualized using nitro blue tetrazolium chloride (NBT) and 5-bromo-4-chloro-3-indolyl phosphate (BCIP) in alkaline phosphate buffer (100 mM NaCl,100 mM Tris pH 9.5, 50 mM MgCl_2_, and 0.5% Tween).

## Supplementary Material

evac172_Supplementary_Data

## Data Availability

Sources of data used in this article are listed in Table 2. All data are publicly available. Phylotocol, custom scripts, command lines, and alignments used in these analyses, and trees resulting from these analyses, are available at https://github.com/josephryan/Steinworth_CnidarianHox. Release v1.0 of this repository, which represents the state of the repo at publication time, has been deposited on Zenodo (doi:10.5281/zenodo.7463438).
