# Supplementary material for: Gene Loss may have Shaped the Cnidarian and Bilaterian Hox and ParaHox Complement
Source: Genome Biol Evol. 2022 Dec 12;15(1):evac172. doi: 10.1093/gbe/evac172 (PMC9825252; doi:10.1093/gbe/evac172)
Supplement: evac172_Supplementary_Data [file evac172_supplementary_data.zip › Steinworth_Suppl_Figs.docx]

**Supplementary Figure Captions**

**Supplementary Figure S1. Bayesian phylogenies of cnidarian and bilaterian Hox and Hox-related genes.** We generated a consensus tree for each of two independent runs of MrBayes, because they did not converge. The tree is rooted using the HlxB9/Mnx clade for display. Newly identified cnidarian homeodomains were named according to the oldest published cnidarian gene in their clade. These gene-defining clades were consistent across both Bayesian trees as well as the maximum likelihood trees generated by RAxML and IQ-TREE. Even with the broadest taxonomic sampling published to date, relationships among these clades vary across the trees and are weakly supported, so we cannot say with confidence whether the trees accurately reflect evolutionary relationships among these genes. Duplicate of Csow_Evx, Cxam_Cnox1, Cxam_111384, Cxam_g9676, and Cxam_g7042 were pruned from the tree in Figure 1 but here are left in, identified with the transciptome or genome number rather than renamed. Colors: black = bilaterian, red = octocorallian, yellow = hexacorallian (excluding ceriantherians), orange = ceriantharian, blue = hydrozoan, green = acraspedan, i.e. non-hydrozoan medusozoan.

**Supplementary Figure S2. Maximum-likelihood phylogeny of cnidarian and bilaterian Hox and Hox-related genes with endocnidozoan *Polypodium hydriforme*.** At the suggestion of a reviewer, we re-ran the phylogenetic analysis with the addition of homeobox sequences from the transcriptome of endocnidozoan *Polypodium hydriforme,* in the interest of thorough taxonomic sampling. *P. hydriforme* sequences are displayed as their transcriptome identification number, which begins with TR. We also added Ro genes from *B. floridae, D. melanogaster,* and *T. castaneum*, which we accidentally omitted from the main analysis. Neither addition changes the overall topology of the tree or our conclusions about cnidarian and bilaterian Hox gene evolution. From the *P. hydriforme* homeobox sequences, we identified Hox-related genes HD065, Gsx, Mox, and Evx, as well as the medusozoan-specific Hox gene we have named Cnox4. Additional *P. hydriforme* sequences formed a clade with no other genes or sequences, implying these genes are not closely related to the rest of the Hox and Hox-related genes in the tree, and we have rooted the tree with this clade for display. Because the *P. hydriforme* sequences are from transcriptomic data, it is possible there are additional Hox or Hox-related genes in the genome, but it is also possible *P. hydriforme* has greatly reduced its Hox complement due to its parasitic lifestyle and corresponding altered body form. The presence of a *P. hydriforme* Cnox4 gene implies this gene arose in the last common ancestor of endocnidozoans and medusozoans, two clades Chang et al. reported as sister groups (Chang et al. 2015). The three bilaterian Ro genes group with the *C. rubrum, N. vectensis,* and *R. reniformis* Ro genes (BS=76). Colors: black = bilaterian, red = octocorallian, yellow = hexacorallian (excluding ceriantherians), orange = ceriantharian, blue = hydrozoan, green = acraspedan, i.e. non-hydrozoan medusozoan.

**Supplementary Figure S1. Bayesian phylogenies of cnidarian and bilaterian Hox and Hox-related genes.**


**Supplementary Figure S2. Maximum-likelihood phylogeny of cnidarian and bilaterian Hox and Hox-related genes with endocnidozoan *Polypodium hydriforme*.**

**Supplementary Table S1. Primers used to clone *Cassiopea xamachana* genes.**

| **Gene name** | **Forward primer** | **Reverse primer** |
| --- | --- | --- |
| CxCnox2a | TTTCAGACCCGTTTGGATACCC | ACTTCGCTGCTTTGCTTCATTT |
| CxCnox4 | GAGGTTGCTTTGCAGAATGGAC | ACCCTTCTGTTTTGAAACCAGGT |
| Cx_g9676 | CTGGTGTTCAAAAACCTAATTTGCC | CATCCAAGTTGCTGATCAGTTCG |
| Cx_g7042 | GCTGTAAAATCATACCCCACTTCAA | GGTTCTCCTTCAATTGTGTTTTCCT |
| CxGsx | GATAAGGCTCAAATTGTCAGCTCC | CGAATTTGGGTATTCGAGATCATTTCC |
| CxHD065 | GGTAACATGCCTATTGAAGACAGTATG | TGAGCATTCAACTTCATCCCTCTC |
| CxHox9-14B | TTGCAGAGGTACAACTTGTCTATCA | GGCGACTATTTTCACACACTGG |
| CxHox9-14C | CATCGTAAGCTCTTATTGTGTCTCTTA | GAGTCTGTTTTGTTCTCTTTCTTCCTC |

These primers were used to clone genes from *C. xamachana* cDNA from mixed stages of embryos, polyps, and medusae.

**Supplementary File S2. RNA extraction protocol for *C. xamachana*.**

Protocol from David A. Gold (davidadlergold.faculty.ucdavis.edu)

1. Cover tissue sample with 200 µl extraction solution (100 mM Tris/HCl pH 5.5, 10 mM EDTA, 0.1 M NaCl, 1% SDS, 1% ß-mercaptoethanol).
2. Add 2 µl RNAse inhibitor.
3. Homogenize tissue sample thoroughly.
4. Add 2 µl Proteinase K, vortex, and incubate at 55°C for 10 minutes.
5. Put on ice, add 11 µl 0.2 M sodium acetate and 250 µl phenol:chloroform: isoamyl alcohol (25:24:1).
6. Vortex for 10 seconds and keep on ice for 15 minutes.
7. Centrifuge for 15 minutes at 12,000 g at 4°C.
8. Transfer upper phase to new tube, add 1 volume isopropanol, and mix well. Keep at -80°C overnight.
9. Centrifuge for 15 minutes at 12,000 g at 4°C.
10. Remove supernatant and wash pellet in 1 ml of 70% ethanol.
11. Centrifuge for 5 minutes at 4°C at 7,500 g.
12. Remove supernatant and air-dry the pellet in a fume hood for 5-10 minutes.
13. Resuspend pellet in 20 µl of nuclease-free water.
14. Protocol can be followed with a commercial RNA cleanup kit for higher quality RNA.
