# Supplementary figures and images for: Gene Loss may have Shaped the Cnidarian and Bilaterian Hox and ParaHox Complement
Source: Genome Biol Evol. 2022 Dec 12;15(1):evac172. doi: 10.1093/gbe/evac172 (PMC9825252; doi:10.1093/gbe/evac172)

**A**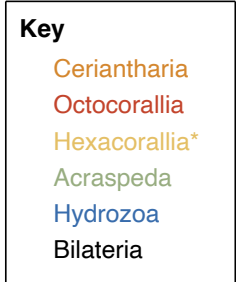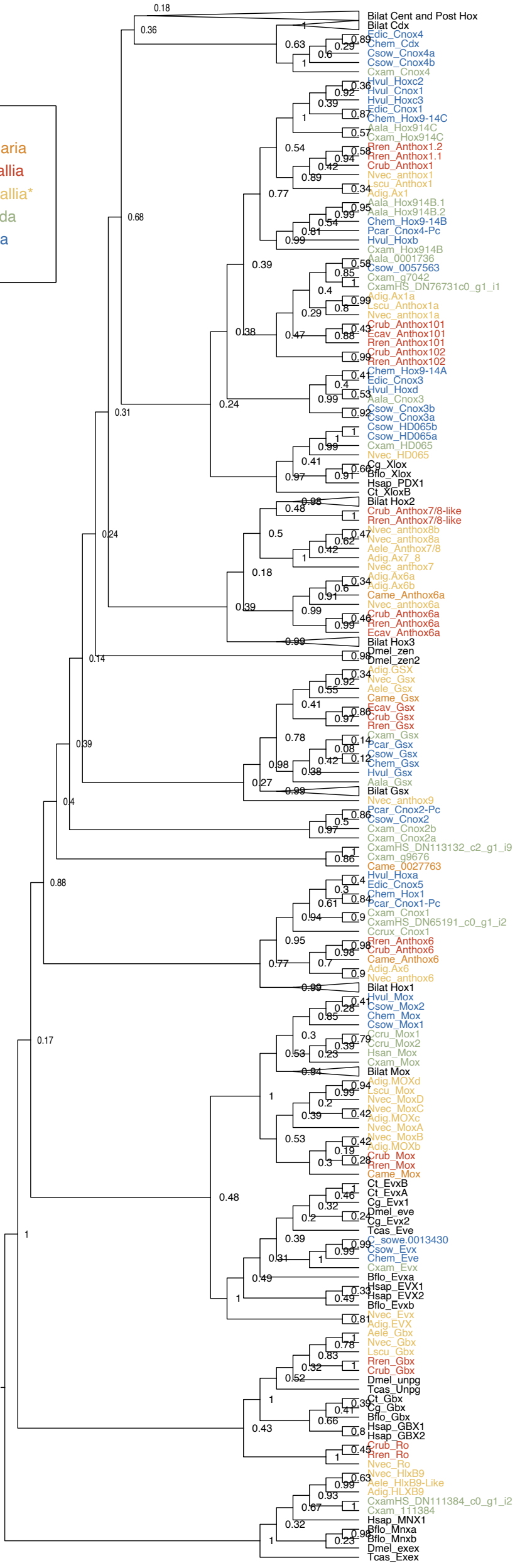**B**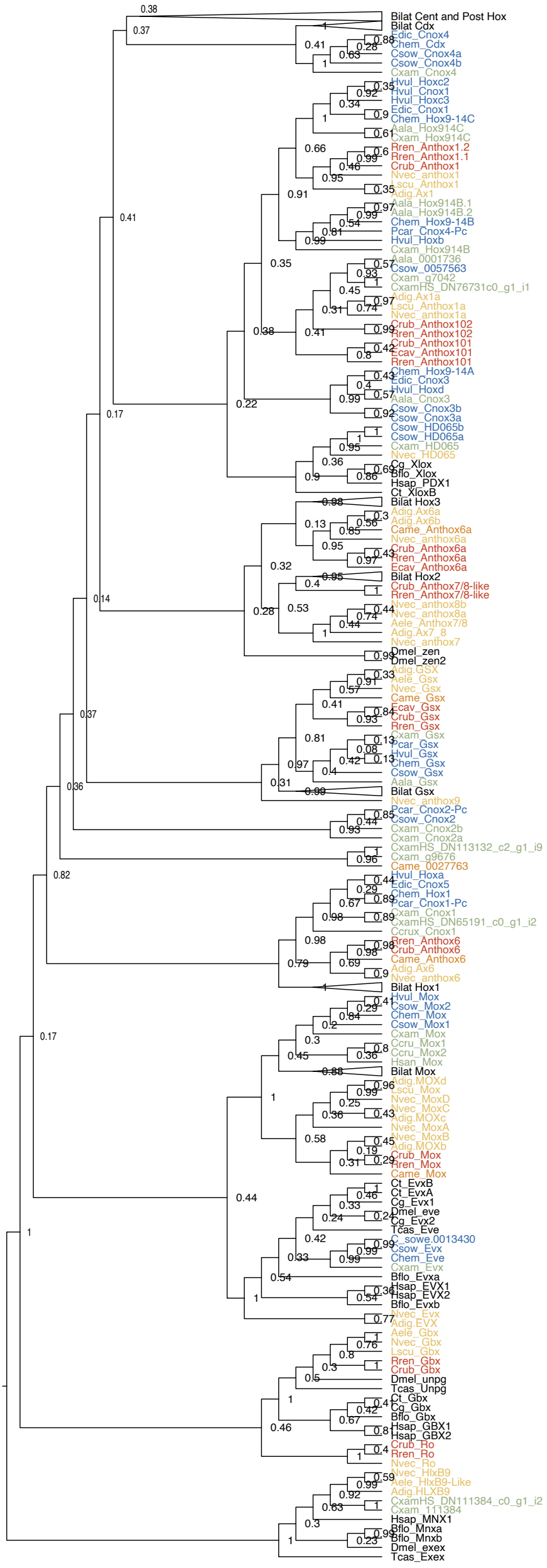

Supplement: evac172_Supplementary_Data [file evac172_supplementary_data.zip › Steinworth_SupplFigS1.pdf]

Key

Polypodiozoa

Ceriantharia

Octocorallia

Hexacorallia\*

Acraspeda

Hydrozoa

Bilateria

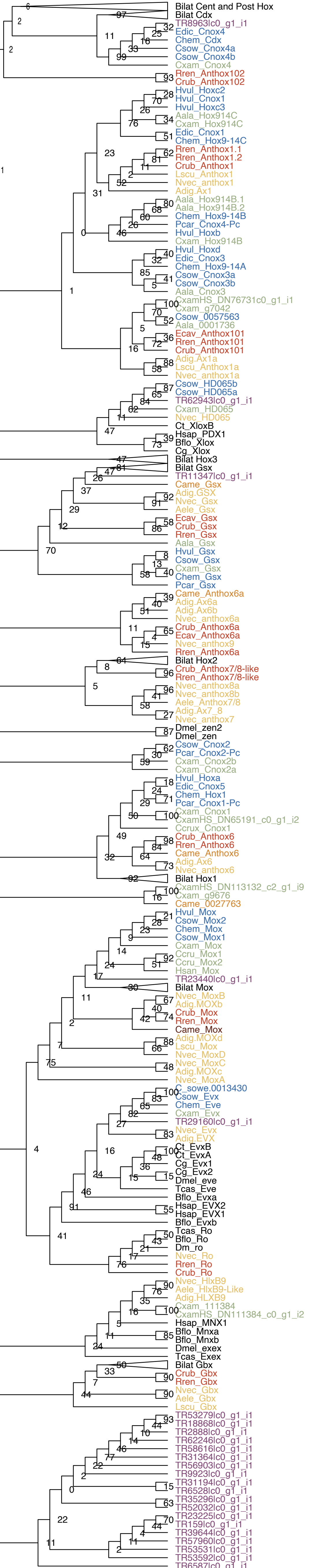

Supplement: evac172_Supplementary_Data [file evac172_supplementary_data.zip › Steinworth_SupplFigS2.pdf]
